# Bronchiolitis obliterans following toxic epidermal necrolysis: a case report

**DOI:** 10.1186/s13256-021-02739-z

**Published:** 2021-03-30

**Authors:** Ujjwal Prakash Khanal, Akash Roy, Arun Kumar Sharma

**Affiliations:** Institute of Medicine, Tribhuwan University, Kathmandu, Nepal

**Keywords:** Toxic epidermal necrolysis, Stevens-johnson syndrome, Bronchiolitis obliterans

## Abstract

**Background:**

Toxic epidermal necrolysis (TEN) is a rare, acute and potentially fatal skin condition usually induced by drugs. Although much attention is focused on the life threatening acute cutaneous and sight threatening ocular manifestations of this disease, chronic pulmonary complications like bronchiolitis obliterans are occasionally encountered. However, little is known about its incidence, pathogenesis, clinical course and outcome in children recovering from TEN.

**Case presentation:**

We report a five-year-old boy who presented four months after the first manifestation of drug-induced TEN with cough and shortness of breath and was subsequently diagnosed with bronchiolitis obliterans. He was treated with supportive therapy that improved his hypercapnia allowing him to be discharged on domiciliary oxygen, chest physiotherapy and bronchodilators.

**Conclusions:**

This case highlights the need to be vigilant for adverse drug reactions and consider chronic pulmonary complications like Bronchiolitis Obliterans in children recovering from TEN.

## Background

Toxic epidermal necrolysis (TEN) is a rare, acute and potentially fatal skin condition usually induced by drugs [1]. It forms a part of a spectrum of blistering skin diseases along with Stevens- Johnson Syndrome (SJS) and erythema multiforme [2]. Children usually present with acute cutaneous manifestations of multiple bullae and skin sloughing resulting from keratinocyte cell death along with mucosal involvement in the oropharynx, eyes and genitals [3]. Respiratory and gastrointestinal mucosa are involved less frequently [1, 4]. Bronchiolitis obliterans (BO) is an extremely rare complication due to chronic involvement of respiratory mucosa; little is known about its incidence, pathogenesis, clinical course and outcome in children recovering from TEN [5].

BO is characterized by bronchiectasis of the large airways along with luminal obstruction and obliteration of the small airways. It is a rare chronic obstructive lung disease in children secondary to an insult to the terminal airway and adjacent lung tissue. The most commonly identified predisposing factor is severe viral infection in the developing lung in children under three years of age [6]. Additionally, there are a number of rare causes of BO and the syndrome is being increasingly identified as a complication of hematopoietic stem cell and lung transplantations [7]. BO secondary to TEN has only been occasionally reported [5, 8, 9].

We present a rare case of a 5 year-old boy with drug induced TEN complicated by bronchiolitis obliterans. To our knowledge, this is the first report of TEN leading to bronchiolitis obliterans in Nepal.

## Case presentation

A previously healthy 5 year-old boy of Indo-Aryan ethnicity with no significant family or psychosocial history had been treated in another hospital for multiple blisters over the trunk, back, limbs and genitals along with marked redness and swelling of the eyes and the mouth 1 day after taking ciprofloxacin orally for suspected enteric fever from a local clinic four months back. Based on the history of drug exposure and typical clinical findings, a diagnosis of drug-induced Toxic epidermal necrolysis was made. During the course of his hospital stay, he developed respiratory failure and required 35 days of intensive care treatment including mechanical ventilation. He showed transient improvement with supportive care and was discharged home after 2 months. During recovery, however, he had vision limiting symblepharon and ankyloblepharon in both eyes along with persistent discharge from both ears.

Four months later, he presented to the emergency room in this hospital with a history of gradually progressive shortness of breath for the past 3 months which had progressed to dyspnea at rest, limiting his daily activities. He had presented after an acute exacerbation of his symptoms with development of a new spell of fever and cough for the past week. On examination, he was visibly distressed with multiple bouts of cough exacerbated on bending over accompanied by production of yellowish sputum. The heart rate was 150 beats per minute, the respiratory rate was 22 breaths per minute, the temperature was 98^0^F, and pulse oximetry recorded an arterial oxygen saturation of 86% in room air. He looked emaciated; he had lost 1.5 kg of weight over the last 2 months. There was no obvious pallor, icterus, or cyanosis, but digital clubbing was prominent.

Further examination revealed significantly restricted chest movements bilaterally with pectus carinatum and signs of significant respiratory distress. Lungs revealed diminished breath sounds bilaterally with prominent wheezing and crackles diffusely. Cardiac auscultation was noncontributory. Several areas of cutaneous hypopigmentation throughout the anterior, lateral and posterior chest wall remained as residual lesions of previous TEN. He was not edematous and other signs of heart failure were absent.

**Fig. 1:**
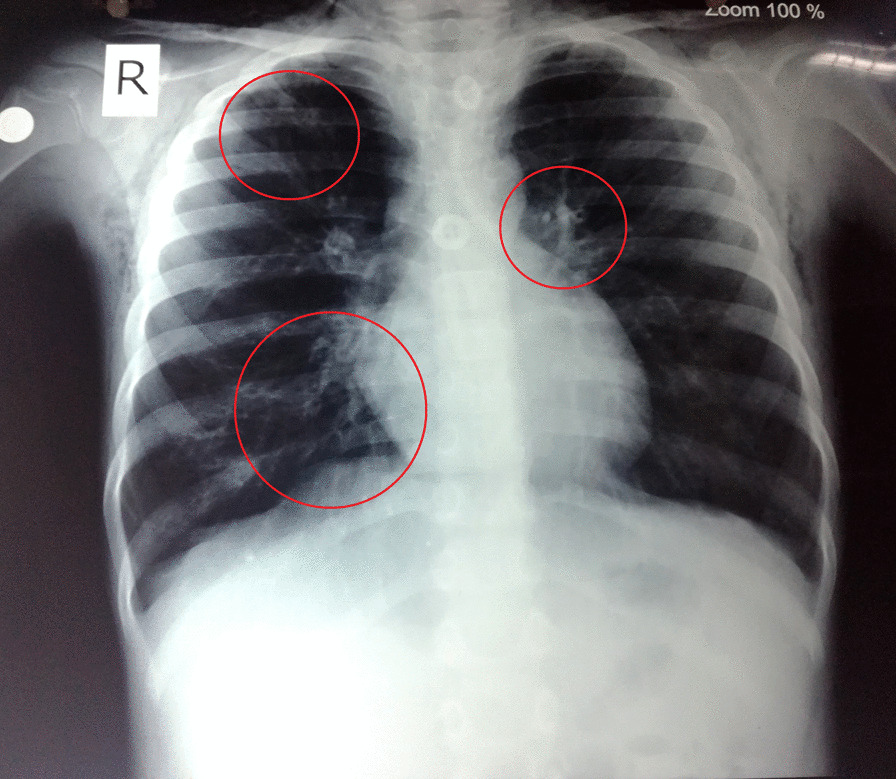
Chest X-Ray showing dilated bronchi in areas circled in red

**Fig. 2:**
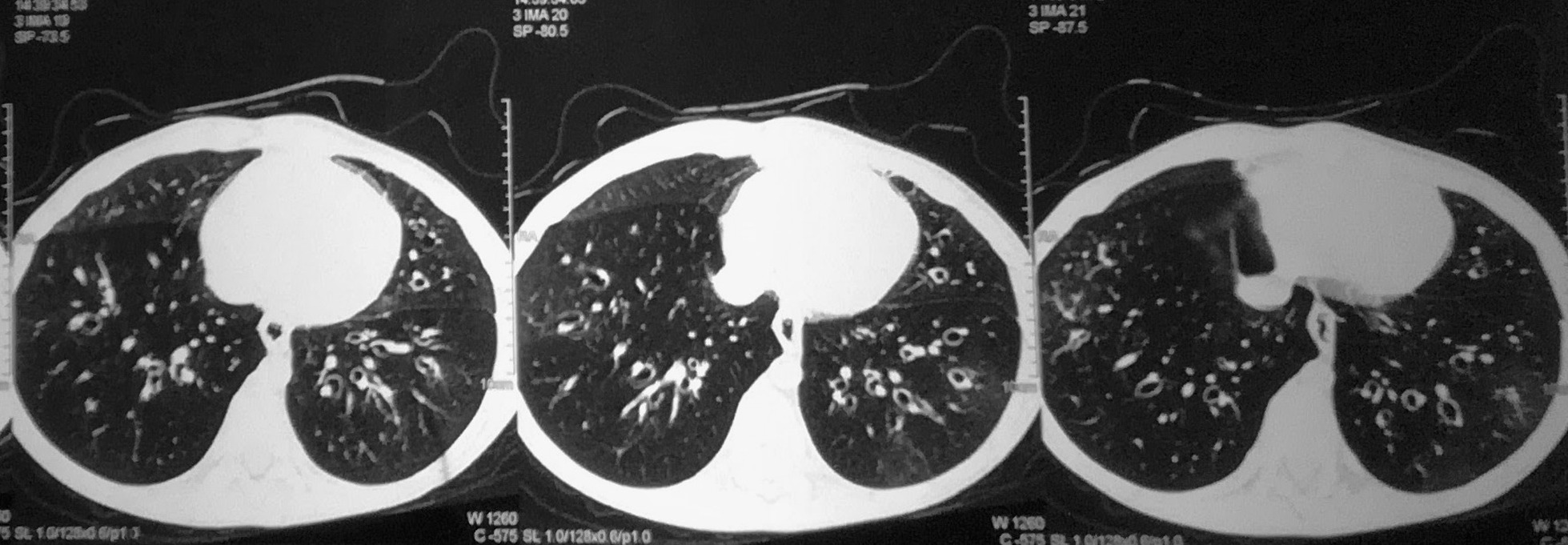
High resolution computerised tomography (HRCT) scan showing bronchiectatic changes in bilateral lower lobes

A blood gas analysis revealed chronic respiratory acidosis. Routine laboratory investigations revealed hemoglobin of 11.8 mg/dl and a blood count of 14,900/µL (66% Neutrophils, 27% lymphocytes, 4% eosinophils and 3% monocytes) with an erythrocyte sedimentation rate (ESR) of 25 mm in first hour. A Chest X-ray revealed dilated bronchi in upper and lower lobes on both right and left lungs (Fig. 1). Renal function parameters were within the normal range. Results for perinuclear anti-neutrophil cytoplasmic antibodies (p-ANCA), cytoplasmic anti-neutrophil cytoplasmic antibodies (c-ANCA) and anti glomerular basement membrane (GBM) antibodies were negative. He could not complete spirometry; pulmonary function testing was inconclusive. Bronchoscopy could not be performed because of persistent hypoxemia. Human immunodeficiency virus serology was non-reactive. A high-resolution computerised tomography (HRCT) scan showed areas of decreased lung attenuation with reduced caliber vessels representing a combination of air trapping and oligemia producing a mosaic attenuation pattern. Patchy ground glass opacities were noted in some areas along with multiple dilated non-tapering tubular bronchi predominantly in the basal segment of the bilateral lower lobes (Fig. 2). The radiological features were consistent with bronchiolitis obliterans.

He was treated for acute exacerbation of his respiratory failure with mechanical ventilation in the PICU and received supportive therapy including bronchodilators, steroids and antibiotics. He required a prolonged hospitalization to be stabilized and could only be discharged home after 2 months. Even at discharge, he continued to remain hypoxemic and was discharged on domiciliary oxygen with frequent chest physiotherapy. At follow up evaluation after 4 weeks, he continued to remain hypoxemic on room air and showed mild respiratory distress even at rest.

## Discussion

SJS/TEN are considered to be more common in children than in adults [10]. Antoon *et al.* reported an incidence of 6.3, 0.7 and 0.5 patients per 100,000 children for SJS, SJS/TEN overlap and TEN respectively in the US population [10]. The commonest known triggers for SJS/TEN are drugs (60-90%), especially antibiotics and anticonvulsants followed by *Mycoplasma* infections. As many as 5–18% of pediatric cases are reported to be idiopathic [11]. Despite lower mortality rates than adults, almost half the children have long term ocular and cutaneous complications [12]. Chronic pulmonary complications like BO are exceedingly rare with only scattered case reports in published literature [9].

Drug induced SJS and TEN occur as delayed-type hypersensitivity reactions. SJS/TEN manifest with "influenza-like" prodromal phase (malaise, fever), followed by cutaneous and mucous membrane (ocular, oral, and genital) eruptions associated with systemic symptoms. Degree of skin involvement differentiates SJS, SJS/TEN overlap, and TEN. SJS is defined as skin involvement of < 10%, TEN is defined as skin involvement of > 30%, and SJS/TEN overlap as 10-30% skin involvement. Diagnosis is established with typical clinical presentation supported by histopathology and specifically immunohistochemistry to rule out other vesicobullous disorders. However, when rapid histopathological and immunohistochemistry facilities are not available, like in many resource poor settings, the diagnosis mainly relies on clinical grounds. [1, 4] The most common drugs linked with SJS/TEN are sulfa groups of antimicrobials, anti-epileptic drugs and non-steroidal anti-inflammatory drugs [13]. HIV is considered an independent risk factor for SJS/TEN with 100 fold increased risk described in the population of HIV positive patients [14].

In the last few years, our understanding of the pathogenesis of SJS/ TEN has improved. Role of drug-specific cytotoxicity mediated by T cells, genetic linkage with HLA and non-HLA genes, TCR restriction, and cytotoxicity have been better clarified as contributory mechanisms to toxic epidermal necrolysis [15]. However, the pathogenesis of BO after TEN is not very well understood [16]. It is characterized by inflammation of subepithelial structures and dysregulated repair, leading to fibro-proliferation and abnormal regeneration of small airway epithelium resulting in concentric narrowing of bronchiolar lumen which may lead to complete luminal occlusion. There are reports of primarily immunologically mediated diseases like rheumatoid arthritis, Sjogren's syndrome and Castleman's lymphoma leading to BO suggesting that this complication is also immune mediated [17, 18]. Secondary infections are also reported to contribute to late pulmonary deterioration [19]. In this case too, the tracheal aspirate was found positive for gram positive cocci.

In addition to SJS/TEN, BO is known to occur following viral infections in young children. With the increasing number of hematopoietic stem cell transplantations (HSCT), BO-like presentation has been identified as an important complication in HSCT and lung transplant recipients as well. Uncommonly, inhalation of toxic fumes, exposure to certain drugs, allergic reactions, collagen disorders and graft versus host disease have been identified to be linked with development of BO syndrome. While acute pulmonary complication requiring mechanical ventilation has been described in about 20% of patients with SJS/ TEN, chronic pulmonary complications like BO are described infrequently [20].

Clinically, BO is characterized by dyspnea and wheezing that are not alleviated by bronchodilators or corticosteroids, severe obstructive pulmonary dysfunction, and obstructive changes in relatively central bronchi that may be confirmed by bronchography and bronchoscopic findings [21]. Spirometry typically reveals an obstructive pattern that does not reverse with bronchodilator challenge. Diffusion capacity (DLCO) is usually reduced. Chest radiographs may be normal in early disease or show signs of hyperinflation. Bronchoscopy reveals central bronchiectasis and obstruction of peripheral bronchi. The HRCT scan shows a mosaic pattern which is a patchwork of low and high density regions (resulting from air trapping by constricted and partially obliterated bronchus) and is now considered a sufficient tool for diagnosis without the need for lung biopsy. Bronchiectasis and bronchiolectasis represent severe scarring and are late findings [22]. Lung biopsy has long been considered gold standard for diagnosis of BO, but heterogeneity of distribution of BO often leads to sampling errors, and may show normal findings in up to 1/3^rd^ of patients [16, 21].

In this case, the exact cause of TEN was not identified but ciprofloxacin consumed prior to onset of TEN was a strong suspect, which has been previously reported to cause severe cutaneous adverse reactions [23]. Subsequently, the patient developed cough with a copious amount of yellowish sputum and shortness of breath one month after onset of TEN that was progressive till this presentation 4 months after the skin manifestation. The clinical picture was consistent with BO but spirometry, bronchoscopy or lung biopsy could not be performed to confirm the diagnosis because of persistent hypoxemia. However, high resolution CT chest showed mosaic pattern along multiple dilated non-tapering tubular bronchi, suggesting bronchiectasis, further supporting the diagnosis of BO.

The treatment of uncomplicated TEN involves supportive care similar to that received by burn patients, including local care of wounds, nutritional support, analgesia and fluid management.[1] While topical steroids are useful for treatment of eczema and allergy, their use in SJS/TEN has not been established yet.[24, 25] However, no effective therapy has been reported once BO is established. Supportive measures with avoidance of tobacco smoke and other inhaled irritants, annual influenza vaccination, chest physiotherapy and airway-clearance, nutritional support, bronchodilators and inhaled corticosteroids are usually helpful. Azithromycin thrice a week and immunomodulatory agents have been tried and have shown promise [21]. Oral azithromycin has been found to be particularly effective in reducing airway neutrophilia in BO [26]. Lung transplantation is considered the only curative therapy for such cases and there have been reports of successful transplantation in children with BO following SJS [19, 21]. In resource poor settings like ours, however, this is not yet a reliable solution.

The prognosis of BO is variable, with many patients having fatal outcomes or surviving with severe airflow obstruction [27]. Compared to post-infectious BO, BO following SJS/TEN has been reported to have a more progressive course [21]. However, a recent review of 22 patients with chronic pulmonary complications following TEN suggested the prognosis may not be as bad as previously considered, with the majority of the patients surviving [9]. In our case, follow-up could only be performed till one month after discharge, where the patient's symptoms had not improved. This case highlights the need to be vigilant for adverse drug reactions and consider chronic pulmonary complications like BO in children recovering from TEN.

## Conclusions

SJS/TEN can lead to chronic pulmonary complications like bronchiolitis obliterans. The diagnosis and definitive management of bronchiolitis obliterans following SJS/TEN are both technically and economically challenging especially in a resource limited setting like Nepal. Therefore, appropriate vigilance after any drug therapy for complications and consideration of bronchiolitis obliterans in children presenting with respiratory symptoms following toxic epidermal necrolysis is necessary.

## Data Availability

All data generated or analysed during this study are included in this published article.

## Abbreviations

TEN: Toxic Epidermal Necrolysis
SJS: Stevens-Johnson Syndrome
BO: Bronchiolitis Obliterans
HIV: Human Immunodeficiency Virus
ICU: Intensive Care Unit
p-ANCA: Perinuclear Anti-Neutrophil Cytoplasmic Antibodies
c-ANCA: Cytoplasmic Anti-Neutrophil Cytoplasmic Antibodies
GBM: Glomerular Basement Membrane
CT: Computerised Tomography
PICU: Pediatric Intensive Care Unit
HLA: Human Leukocyte Antigen
DLCO: Diffusing capacity of Lung for Carbon Monoxide
HRCT: High Resolution Computerised Tomography
